# Determinants of impaired quality of life in patients with fibrous dysplasia

**DOI:** 10.1186/s13023-017-0629-x

**Published:** 2017-04-27

**Authors:** Bas C. J. Majoor, Cornelie D. Andela, Jens Bruggemann, Michiel A. J. van de Sande, Ad A. Kaptein, Neveen A. T. Hamdy, P. D. Sander Dijkstra, Natasha M. Appelman-Dijkstra

**Affiliations:** 1 Department of Orthopaedic Surgery, Center for Bone Quality Leiden University Medical Center, Albinusdreef 2, Postzone J11, PO Box 9600, 2300 RC Leiden, The Netherlands; 2 Department of Medicine: Division Endocrinology, Center for Bone Quality Leiden University Medical Center, Albinusdreef 2, Postzone J11, PO Box 9600, 2300 RC Leiden, The Netherlands; 3 Department of Medical Psychology, Center for Bone Quality Leiden University Medical Center, Albinusdreef 2, Postzone J11, PO Box 9600, 2300 RC Leiden, The Netherlands

**Keywords:** Fibrous dysplasia, McCune-Albright syndrome, Quality of life, Pain, Biochemical markers of bone turnover

## Abstract

**Background:**

Fibrous dysplasia is a rare bone disorder, commonly associated with pain, deformity and fractures, which may significantly impact on quality of life. In this study we evaluate quality of life in patients with fibrous dysplasia using the Short Form-36 and the Brief Pain Inventory questionnaires. Data were compared with those of the general Dutch population.

**Results:**

Out of 138 patients from a cohort of 255 patients with fibrous dysplasia that were sent questionnaires assessing quality of life and pain, the response rate was 70.3%, with 97 patients, predominantly female (65%), completing the questionnaires. Monostotic fibrous dysplasia was predominant (*n* = 62, 64%). Fibrous dysplasia patients had significantly lower quality of life outcome scores than the general Dutch population for all tested domains of the Short Form-36 except for the “Mental health” and the “Role emotional” domains. More severe forms of fibrous dysplasia, had the more severe Short-Form-36 quality of life outcomes, but there was no significant difference in Brief Pain Inventory domains between different subtypes of fibrous dysplasia. Quality of life was lower in patients with higher disease burden, as reflected by high skeletal burden scores (*p* = 0.003) and high levels of P1NP (*p* = 0.002).

**Conclusion:**

We demonstrate impairments in all domains of quality of life, except for ‘Mental health’ and ‘Role emotional’ domains, across the wide spectrum of fibrous dysplasia including its milder forms. We identified high skeletal burden scores, reflecting disease severity, as the most consistent predictor of impaired quality of life. Our findings hold significant clinical implications as they draw attention to the clinically unmet need to address quality of life issues in the management of patients with all subtypes of fibrous dysplasia, including its milder forms.

## Background

Fibrous dysplasia (FD) is a rare congenital bone disorder, caused by a missense mutation of the GNAS-gene [1]. The bony lesions may involve one bone (monostotic FD), or multiple bones (polyostotic FD), and be associated with extra skeletal manifestations such as endocrinopathies (precocious puberty and growth hormone excess) and/or café-au-lait patches in McCune-Albright syndrome (MAS), or intramuscular myxomas in Mazabraud syndrome. The disorder often manifests itself in childhood, presenting with bone pain, deformities or a pathological fracture, although the disease may be also asymptomatic, with bony lesions being incidentally identified on radiographic imaging [2]. The clinical spectrum of FD is thus very broad, varying from the mild asymptomatic single bone lesion in the monostotic forms of the disease, to the potentially crippling polyostotic forms, with or without additional extraskeletal manifestations, which may considerably impact on various aspects of quality of life.

Quality of life (QoL) is defined as “the functional effect of an illness and its consequent therapy upon a patient, as perceived by the individual patient” [3] and may thus significantly vary between individual patients with the same disease. QoL can be assessed by the use of generic questionnaires, such as the Short-Form 36 (SF-36) questionnaire, or by disease-specific or domain-specific questionnaires, including questionnaires on symptoms associated with the condition under study such as the Brief Pain Inventory (BPI) to assess pain [4, 5]. Data on QoL are scarce in FD. Physical function scores have been reported to be lower in 56 adult patients with polyostotic FD than those reported in the general US population [3]. The same study identified a direct relationship between “Physical function” scores and skeletal burden scores (as calculated from Tc-99m skeletal scintigraphy images), which reflect disease-extent and thus severity. However, Social and Emotional domains were not affected by disease severity, illustrating the relative independence of ‘objective’ severity of a medical condition and its effects on the quality of life of patients [6].

The aim of our study was to evaluate QoL in a large cohort of well-characterized FD patients followed up at the Center for Bone Quality of the Leiden University Medical Center for up to 25 years. A further aim of our study was to evaluate whether patients with the different subtypes of FD (monostotic FD, polyostotic FD, or MAS) are differentially affected in their QoL. We hypothesized that patients with MAS, with the more extensive skeletal lesions and endocrinopathies, would exhibit more impaired QoL compared to the less severely affected patients with monostotic and polyostotic FD. A last aim of our study was to assess potential factors contributing to impaired QoL, including extent of skeletal burden, presence and severity of pain and biochemical parameters of bone turnover as potentially reflecting FD disease activity.

## Methods

**Fig. 1:**
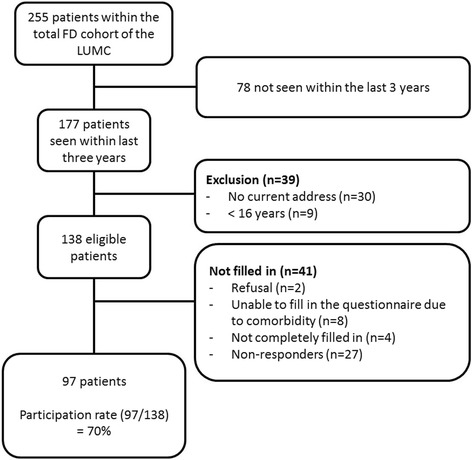
Flowchart of patient inclusion

Patients recruited for this study belonged to our Center’s well-characterized cohort of patients with FD, which consists of 255 patients with a full spectrum of FD subtypes: monostotic, polyostotic, MAS and Mazabraud syndrome [7]. Patients were invited to participate in this cross-sectional study on the basis of the following criteria: age16 years or older who had a fixed current address (to insure that they could be reached), and who had been seen at least once at our outpatient clinic within the preceding three years. These inclusion criteria were fulfilled by 138 patients who were sent letters to their registered home addresses, inviting them to complete the QoL questionnaires (Fig. 1). Patients who failed to respond to our letter of invitation were contacted by telephone.

Two patients refused to take part in the study, 8 patients were unable to complete the questionnaires because of non-FD related comorbidities, and 27 patients who did not respond to our letter of invitation and could also not be contacted by telephone. There were no significant differences in age, type of FD or skeletal burden scores between patients who responded (*n* = 97) and those who did not, but more women responded than men (*p* = 0.039).

The protocol was approved by the Medical Ethics Committee of the LUMC and written informed consent was obtained from all patients who completed the questionnaires.

### Clinical and sociodemographic characteristics

The diagnosis of FD was established in all patients on the basis of clinical and radiological features, occasionally requiring additional histologic confirmation. Sociodemographic parameters were retrieved from the patients’ electronic medical records. Level of education was determined on the basis of the International Standard Classification of Education (ISCED). A low level of education was defined as a primary to lower secondary education; a medium level of education was defined as an upper secondary to post-secondary non-tertiary education; and a high level of education was defined as the first and second stage of tertiary education. Data on age, gender, age at diagnosis, level of education, type of FD, extra-skeletal manifestations (e.g. precocious puberty, GH-excess, café-au-lait patches), prevalence of fractures and details of therapy, surgical and/or medical, were also documented. Data on biochemical parameters of bone turnover were also retrieved from the patients’ records when obtained within two months before or after completing the questionnaires. These included data on total alkaline phosphatase (ALP), as measured by a colorimetric method on the Roche Modular P800 analyser from Roche Diagnostics, Almere, The Netherlands, and on procollagen 1 amino-terminal propeptide (P1NP) and beta crosslaps (CTX), both measured using an electrochemoluminescent immunoassay with a Modular Analytics E-170 system (Roche Diagnostics, Almere, The Netherlands. Data were also retrieved for C-terminal FGF-23 (Immutopics, San Clemente, CA, USA) as measured using the BioTek ELx50 Washer (BioTek, Bad Friedrichshall, Germany) after short storage at −20°C prior to analysis [8]. Two authors (BCJM and NMA-D), who were blinded to the identity of the patients, evaluated the ^99^Technetium skeletal scintigraphic images, when performed within the three years preceding entry in the study, for calculation of skeletal burden scores (SBS). Differences in scores were resolved by consensus [9]. In the analysis of potential factors impacting on QoL, we differentiated between constant factors such as SBS, shown not to alter after skeletal growth is completed, and potentially variable factors such as circulating levels of biochemical markers of bone turnover and FGF-23.

### Questionnaires

#### Short form-36

The SF-36 questionnaire has been shown to be a reliable instrument for the evaluation of various domains of QoL in individuals older than 14 years [4]. In this study, we used all collected domain scores of this validated questionnaire to evaluate various aspects of QoL and data were compared with reference scores of the general Dutch population (*n* = 1742) [10].

#### Brief pain inventory

The BPI is an assessment tool that was originally designed to assess pain in patients with cancer [5]. This tool is now also validated in the assessment of non-oncological pain and has been widely used in the protocol of a number of clinical trials evaluating pain of different pathophysiologies [11–13]. Domain scores of the BPI include ‘Pain severity’ and ‘Pain interference’, which were both used as outcome parameters for our study.

#### Statistical analysis

Statistical analysis was performed using SPSS for Windows, Version 23.0 (SPSS, Inc., Chicago, IL, USA). Results are presented as mean (±SD) or as median (intermediate range) and in case of categorical data as a percentage. SF-36 domain scores were compared with the SF-36 reference scores of the Dutch population by using pooled T-tests. Outcomes of the SF-36 and BPI domain scores were compared between different types of FD (monostotic/polyostotic/MAS) using an ANOVA test, with post hoc analysis applied when appropriate. Possible predictors for impaired QoL such as skeletal burden score and biochemical markers of bone turnover at time of completing the questionnaire were assessed with both univariate and multivariate linear regression analysis. Level of significance was set at *p* ≤ 0.01 to correct for multiple testing.

## Results

### Patients’ characteristics (Table 1)

**Table 1:** Patient characteristics

	(*n* = 97)
Gender (Male/Female)	34/63
Age	46 (16–80)
Educational Level	Low 10 (10%)
	Medium 24 (25%)
	High 46 (47%)
	Unknown 17 (18%)
Type of FD	Monostotic 62 (64%)
	Polyostotic 26 (27%)
	McCune-Albright 9 (9%)
	Mazabraud 5 (5%)
Follow-up (years)	12 (0–62)

Data are median (range) or number and percentage

Response rate was 70.3%, with 97 patients, predominantly female patients (*n* = 63, 65%), completing the questionnaire. Median age at diagnosis was 29 years (range 1–68 years), and median age at completion of the questionnaire was 46 years (range 16–80 years). Median duration of follow-up after diagnosis of FD was 12 years (range 0–62 years). Level of education was ‘low’ in 10 patients, ‘medium’ in 24 patients and ‘high’ in 46 patients. Data on level of education were missing in 17 patients. Patients included in the study had predominantly monostotic FD (*n* = 62, 64%), 26 had polyostotic disease (27%), 9 patients had MAS (9%) and five had Mazabraud syndrome (5%). Mean skeletal burden score was 8.68 ± 12.40 SD. Fifty-six patients (58%) had received bisphosphonate treatment at some stage before completion of the questionnaires. There were significant differences in SBS (*p* < 0.001), average FGF-23 (*p* = 0.002), prevalence of at least one fracture (*p* < 0.001) and a history of surgery (*p* < 0.001) between the various types of FD. There was, thus a consistent trend towards more severe QoL outcomes in the more severe forms of FD, with poorer outcomes observed in MAS compared to polyostotic FD, and in polyostotic compared to monostotic FD.

### SF-36 and BPI outcomes

**Fig. 2:**
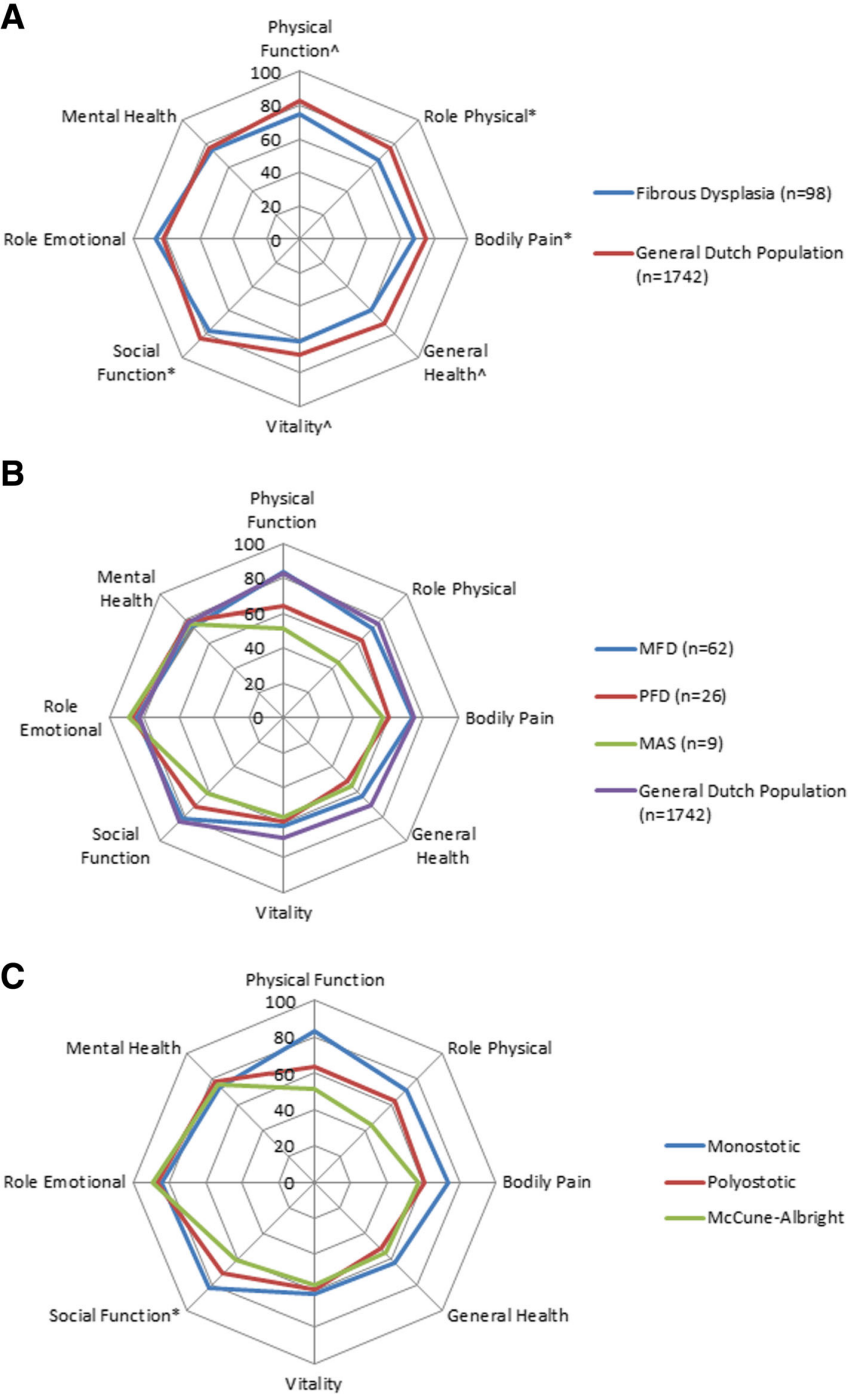
Radar charts comparing the QoL between FD patients and the general Dutch population (**a**), subtypes of FD and the general Dutch population (**b**) and differences between de subtypes of FD (**c**). Significant differences are illustrated by ^*p* < 0.05 or *p < 0.001

Patients with FD had significantly lower QoL outcome scores than the general Dutch population for all domains of the SF-36 except for the “Mental health domain” and the “Role emotional domain” (Fig. 2a) [10]. Outcomes were thus significantly worse in FD compared to the general population for the “Physical Functioning domain” (75 *vs*. 83, *p* < 0.001), “Role Physical domain” (66 *vs*. 76, *p* = 0.007), “Bodily Pain domain” (68 *vs*. 75, *p* = 0.007), “General Health domain” (59 *vs*. 71, *p* < 0.001), “Vitality domain” (61 *vs*. 69, *p* < 0.001) and “Social Functioning domain” (77 *vs*. 84, *p* = 0.004). Compared to the general population, MFD patients had significant impairments in “General Health” and “ Vitality”, PFD patients in “Physical Function”, “Bodily Pain”, “General Health”, “Vitality” and “Social Function” and lastly, patients with MAS had significant impairments in all domains except for “Vitality”, “Role Emotional” and “Mental Health” (Fig. 2b and Tables 2 and 3).

**Table 2:** Comparison of SF-36 scores between subgroups of fibrous dysplasia and the general population

SF-36	Monostotic *n* = 62	Polyostotic *n* = 26	McCune-Albright *n* = 9	General population *n* = 97
Physical Function	83.1 (20)	63.9 (26)^β^	51.1 (34)^β^	83.0 (22)
Role Physical	71.7 (39)	63 (44)	44.4 (39)^β^	76.4 (36)
Bodily Pain	73.9 (25)	60.5 (27)^β^	57.1 (25)^α^	74.9 (23)
General Health	63.1 (23)^β^	52 (22)^β^	55.6 (30)^α^	70.7 (20)
Vitality	62.2 (18)^α^	59.4 (19)^α^	57.2 (16)	68.6 (19)
Social Function	82.3 (21)	71.5 (28)^β^	61.1 (33)^β^	84.0 (22)
Role Emotional	84.3 (31)	87.5 (29)	88.9 (24)	82.3 (32)
Mental Health	73.6 (18)	77.7 (14)	75.6 (10)	76.8 (17)

Data are mean (SD)

^α^ *P* < 0.05 compared to the general population

^β^ *P* < 0.01 compared to the general population

**Table 3:** Comparison of SF-36 scores between subgroups of fibrous dysplasia

SF-36	Monostotic *n* = 62	Polyostotic *n* = 26	McCune-Albright, *n* = 9
Physical Function	83.1 (20)^β,γ^	63.9 (26)^α^	51.1 (34)^α^
Role Physical	71.7 (39)	63 (44)	44.4 (39)
Bodily Pain	73.9 (25)^β^	60.5 (27)^α^	57.1 (25)
General Health	63.1 (23)	52 (22)	55.6 (30)
Vitality	62.2 (18)	59.4 (19)	57.2 (16)
Social Function	82.3 (21)^γ^	71.5 (28)	61.1 (33)^α^
Role Emotional	84.3 (31)	87.5 (29)	88.9 (24)
Mental Health	73.6 (18)	77.7 (14)	75.6 (10)

Data are mean (SD)

^α^ *P* < 0.05 compared to Monostotic

^β^ *P* < 0.05 compared to Polyostotic

^γ^ *P* < 0.05 compared to McCune-Albright

Subgroup analysis yielded a further significant difference between the subtypes of FD for the Physical Functioning (*p* < 0.001), Social Functioning (*p* = 0.016) and Bodily Pain (*p* = 0.015) domains, with increasingly lower QoL scores observed in the more severe types of FD (Fig. 2c and Tables 2 and 3). Patients with MAS demonstrated particularly low scores for the Physical Function (51.1 ± 34) and Role Physical (44.4 ± 39) domains, and both MAS and PFD patients had significantly lower scores for the Bodily Pain (respectively 57.1 ± 25 and 60.5 ± 27), General Health (respectively 55.6 ± 30 and 52.0 ± 22) and Vitality (respectively 57.2 ± 16 and 59.4 ± 19) domains compared to scores in these domains in patients with monostotic FD. Scores did not significantly differ between subtypes of FD in the other 5 SF-36 domains, or in both BPI domains, although polyostotic FD and MAS patients had consistently higher scores than monostotic FD patients in both BPI domains (Table 4).

**Table 4:** Comparison of BPI scores between subgroups of FD

BPI	Monostotic, *n* = 62	Polyostotic, *n* = 26	McCune-Albright, *n* = 9	Total, *n* = 97
Pain Severity	3.0 (2.7)	3.9 (2.7)	4.1 (1.9)	3.4 (2.6)
Pain Interference	2.3 (2.6)	3.1 (2.8)	1.0 (0.8)	2.3 (2.6)

Data are mean (SD)

^α^ *P* < 0.05 compared Monostotic FD

^β^ *P* < 0.05 compared Polyostotic FD

^γ^ *P* < 0.05 compared with McCune-Albright

### Clinical predictors of impairment in QoL

Univariate regression analysis identified female gender (β = -17.6; *p* = 0.002), high SBS (β = -1.08; *p* < 0.001), high serum concentrations of FGF-23 (β = -0.15; *p* = 0.01) and of P1NP (β = -0.05; *p* = 0.001) as significant predictors for impaired Physical Function. High SBS was also associated with low Social Function (β = -0.65; *p* = 0.01) and high levels of P1NP with impaired General Health (β = 0.03; *p* = 0.01) and impaired Role Emotional (β = 0.06; *p* = 0.001). Age and serum levels of ALP and CTX were not associated with any of the QoL domains in univariate regression analysis (Table 5).

**Table 5:** Attributive factors in SF-36 domains

	SBS		FGF-23		ALP		P1NP		CTX	
SF-36 Domains	*R*	*Sig.*	*R*	*Sig.*	*R*	*Sig.*	*R*	*Sig.*	*R*	*Sig.*
PF	*−0,51*	*<0,001*	*−0,49*	*<0,001*	−0,11	0,431	−0,08	0,583	−0,11	0,445
RP	−0,18	0,172	−0,20	0,175	0,01	0,577	−0,06	0,692	−0,01	0,992
BP	−0,11	0,365	−0,22	0,117	0,13	0,328	0,10	0,433	0,07	0,600
GH	−0,01	0,978	−0,22	0,112	0,03	0,835	−0,05	0,700	0,02	0,892
VT	0,03	0,839	−0,13	0,374	0,19	0,147	−0,07	0,628	−0,09	0,516
SF	0,32	0,316	−0,20	0,159	−0,15	0,245	−0,03	0,807	−0,02	0,861
RE	−0,01	0,956	−0,15	0,313	−0,04	0,766	−0,32	0,018	−0,15	0,279
MH	0,21	0,111	−0,07	0,639	0,19	0,180	0,09	0,518	0,03	0,837

Multiple regression analysis was performed including age, gender, P1NP and SBS. FGF-23 and ALP were excluded, as these parameters are known to be respectively correlated with SBS and P1NP [7, 9]. Both high SBS (β = -0.82; *p* = 0.003) and high levels of P1NP (β = -0.04; *p* = 0.002), but not female gender, remained significant predictors for impaired physical function as evaluated by SF-36 (Table 5). High serum levels of P1NP still predicted impaired Role Emotional (β = -0.06; *p* < 0.001), but no longer General Health (β = -0.03; *p* = 0.012) as shown in univariate analysis. Other SF-36 and BPI domains were not affected by any of the factors studied in the multivariate analysis (Table 5 and 6).

**Table 6:** Attributive factors in BPI

	SBS		FGF-23		ALP		P1NP		CTX	
BPI Domains	*R*	*Sig.*	*R*	*Sig.*	*R*	*Sig.*	*R*	*Sig.*	*R*	*Sig.*
Worst pain 24 h	0,22	0,074	0,16	0,288	−0,15	0,281	0,13	0,360	0,07	0,636
Average pain	0,13	0,323	0,20	0,179	−0,27	0,048	0,11	0,410	0,15	0,264
Pain at completion of BPI	0,01	0,961	0,11	0,454	−0,25	0,079	−0,10	0,489	0,07	0,620
Pain Severity	0,13	0,315	0,15	0,314	−0,30	0,035	0,02	0,887	0,04	0,799
Pain Interfrence	0,07	0,592	0,07	0,659	−0,15	0,288	0,12	0,408	21,00	0,397

## Discussion

In this study we demonstrate that patients with FD report significantly impaired quality of life in all tested domains except for Mental Health and Role Emotional. Our data further show that the reported impairment in quality of life is greater in patients with the higher disease burden, as reflected by high SBS and increased concentrations of biochemical markers of bone turnover in the more severe subtypes of FD. As hypothesized, patients with polyostotic FD and MAS had a more pronounced impairment of QoL compared to patients with monostotic FD, likely to be due to their greater risk of developing complications such as deformities and/or fractures [14]. This premise is further supported by the association of higher SBS with higher fracture risk and consistent need for surgical intervention in the more severely affected patients with MAS. In addition to the skeletal complications related to the high skeletal burden, patients with MAS also have one or more endocrinopathies, also shown to be associated with impaired QoL in their own right, which at least partially explains the significantly low scores in physical and social functions domains observed in these patients [15]. In keeping with findings from Kelly et al. we were unable to detect differences in BPI domains between different subtypes of FD, although patients with MAS or polyostotic FD demonstrate a trend toward higher pain scores in both domains [16]. The mental health and role emotional domains were interestingly not affected in any subtype of FD, suggesting preserved psychological function and adequate emotional adaptation in patients suffering from FD, with the diagnosis likely to have been established in childhood.

Data on impaired Physical Function, Role-Physical, Bodily Pain and General Health scores have previously been reported in a US FD cohort [6]. In contrast with findings in this cohort study, we observe a significant impairment in Vitality scores, as expressed by feelings of tiredness and low-energy, and in Social Function scores, as reflected by the degree of interference with social activities due to physical or emotional problems. Our data suggest that patients with FD experience a decrease in energy level and a general feeling of tiredness compared to the general population, potentially precluding or decreasing their participation in social activities. This discrepancy in outcome between the two cohorts may be partially explained by a difference in cohort characteristics between studies, such as the age or gender distribution of the participants. The median age of our cohort was thus 46 years (range 16–80 years), compared to the younger median age of 34.7 years (range 14–86) in the US cohort and older age has indeed been consistently associated with increasing impairments in QoL [3]. Gender distribution might also play a role, as women have reported lower QoL scores in the Dutch population, but also because MAS, the more severe subtype of FD, is more often diagnosed in women in our cohort. [10] Although in univariate regression analysis female gender appeared to be associated with impaired physical function, multivariate analysis revealed no association between gender and any impairments in QoL in our cohort. Nevertheless, we should take into account that this might be the result the sample size of our cohort, as the cohort study showing these impairments in women accounted over 1,700 persons. Different subtype composition of the two cohorts and management differences, including different medical and surgical approaches, may also have played a role in the discrepancy in outcomes between cohorts.

Our data on QoL outcomes in FD are in keeping with those reported in Paget’s disease of bone, a similar benign bone disorder also associated with clinical manifestations of bone pain, deformity and increased risk for pathological fractures. Patients with Paget’s disease of bone have thus also been shown to demonstrate significant reductions in all SF-36 domains, except for the Mental Health domain, suggesting that although benign bone disorders such as Paget’s disease of bone and FD do impact on several aspects of QoL, patients appear to be generally able to mentally cope with their QoL impairments [17].

In addition to confirming the inverse relationship between skeletal burden scores and physical function, our data also show a negative association between FGF-23 concentrations and the SF-36 Physical Function domain [6, 7, 9]. The relationship between SBS, a reliable parameter of FD disease extent and thus severity, and FGF-23 concentrations has been previously demonstrated [7, 9]. SBS has also been shown to be a constant feature of the disease after the growth period, and to alter little or not at all after therapeutic interventions. Taken together, these findings suggest that SBS may be considered to be a reliable predictor for impaired physical function in patients with FD. In contrast, biochemical markers of bone turnover, although also found to be correlated with impaired Physical Function and Role-emotional domains, may prove to be less reliable predictors of impaired physical function as they demonstrate significant variation during the natural course of the disease, which is characterized by periods of activity and remission. These markers are also clearly affected by therapeutic interventions such as the use of bisphosphonates [7].

Our study has some strengths as well as limitations. Of its main strengths are the relatively large number of patients belonging to a well-characterized cohort included in the study, with good representation of the so far ill-described milder monostotic subtype of FD, and the high response rate of 70.3% of patients invited to take part in the study. A further strength of our study is the opportunity to compare our QoL data in FD with QoL data from the general Dutch population.

The main limitation of our study is shared by all studies in rare and heterogeneous diseases, and a further limitation in FD is the still unclear natural history, particularly that of its milder subtypes. A further limitation of our study is its cross-sectional design, with a single measurement of quality of life parameters undertaken at one point in time in patients with FD of various degrees of severity. Notwithstanding, whereas it is generally accepted that multiple sequential measurements of QoL may be more informative on the impact of the disease on various aspects of quality of life, we believe our single measurement data are still very informative, as they not only show the expected impairment of QoL in patients with the more severe polyostotic disease, with or without endocrinopathies, but also demonstrate a sizable impact of the milder monostotic type of the disease on quality of life.

## Conclusion

In conclusion, data from our cross-sectional study demonstrate impairments in all SF-36 domains of quality of life except for the Mental health and Role emotional domains in a relatively large number of patients with a wide spectrum of FD disease severity including its milder forms. We demonstrate that a high skeletal burden score as reflecting disease severity represents the most consistent predictor of impaired QoL. Our findings from this study hold significant clinical implications as they draw the treating physician’s attention to the important clinically unmet need to address quality of life issues in the management of all subtypes of FD, including its milder forms. Whether quality of life can be improved by medical or surgical interventions remains to be established by long-term studies in a large number of patients.

## Abbreviations

BPI: Brief pain inventory
CTX: Beta crosslaps
FD: Fibrous dysplasia
FGF-23: Fibroblast growth factor 23
MAS: McCune-Albright syndrome
P1NP: Procollagen 1 amino-terminal propeptide
SBS: Skeletal burden score
SF-36: Short form 36
